# Neuroendocrine and squamous cell phenotypes of NUT carcinoma are potential diagnostic pitfalls that discriminating it from mimickers, such as small cell and squamous cell carcinoma

**DOI:** 10.1186/s13000-024-01448-7

**Published:** 2024-02-07

**Authors:** Hironori Ninomiya, Yukiko Sato, Kentaro Inamura, Akito Dobashi, Kengo Takeuchi, Hiroki Mitani, Mingyon Mun, Makoto Nishio, Yuichi Ishikawa

**Affiliations:** 1 Division of Pathology, Cancer Institute, Japanese Foundation for Cancer Research, Tokyo, Japan; 2 Department of Pathology, Cancer Institute Hospital, Japanese Foundation for Cancer Research, Tokyo, Japan; 3 Pathology Project for Molecular Targets, Cancer Institute, Japanese Foundation for Cancer Research, Tokyo, Japan; 4 Division of Head and Neck Surgery, Cancer Institute Hospital, Japanese Foundation for Cancer Research, Tokyo, Japan; 5 Thoracic Surgical Oncology, The Cancer Institute Hospital, Japanese Foundation for Cancer Research, Tokyo, Japan; 6 Thoracic Medical Oncology, The Cancer Institute Hospital, Japanese Foundation for Cancer Research, Tokyo, Japan; 7 Department of Pathology, Mita Hospital, International University of Health and Welfare, Tokyo, Japan

**Keywords:** NUT carcinoma, Neuroendocrine marker, INSM1, Synaptophysin

## Abstract

**Introduction:**

NUT carcinoma is a rare cancer associated with a poor prognosis. Because of its rarity, its diagnosis is challenging and is usually made by excluding other diagnoses. Immunohistochemical analysis is a reliable technique that contributes to a correct diagnosis, but overestimating the expression of neuroendocrine (NE) markers may result in an incorrect diagnosis. In this study, we established the immunohistochemical phenotypes of NUT carcinoma compared with tumors that mimic its phenotype to identify potential diagnostic pitfalls.

**Methods:**

Eight cases of NUT carcinoma were examined along with eight basaloid squamous cell carcinomas and thirteen cases of small cell carcinoma using an immunohistochemical panel consisting of various antibodies.

**Results:**

Of the eight NUT carcinomas, three patients had a smoking history. All the cases examined for INSM1 were positive (6/6, 100%), although the staining was somewhat weak. Among the NE markers, synaptophysin was variably positive in two NUT carcinomas (2/6, 33%); however, all cases were negative for ASCL1, chromogranin A, and CD56. Moreover, the squamous cell markers, p40 and CK5/6, were weakly expressed in 4/6 (67%) and 3/6 (50%) of the NUT carcinomas, respectively.

**Conclusions:**

For tumors with an ambiguous morphology, applying the neuroendocrine phenotype of NUT carcinoma may be misleading; particularly, when distinguishing it from small-cell carcinoma. Similarly, null or weak expression of squamous cell markers may be observed in NUT carcinoma, but this differs from squamous cell carcinoma, which consistently demonstrates strong positivity for squamous cell markers.

## Introduction

NUT carcinoma is a highly aggressive, poorly differentiated tumor hallmarked by a *NUT* gene rearrangement. Fusion partners include the bromodomain family members, *BRD3* and *BRD4* [1]*,* or the methyltransferase *NSD3* [2]. Most tumors occur in the thoracic, head, and neck regions [3, 4]. Pathologically, NUT carcinoma typically appears as sheets and nests of small- or intermediate-sized undifferentiated cells exhibiting a monomorphic appearance [1, 5], and occasionally contains a squamous cell component. A primitive morphological appearance can complicate the recognition as NUT carcinoma, particularly if there is a lack of characteristic squamous differentiation, such as abrupt keratinization. Immunohistochemical studies have facilitated rapid diagnosis by enabling the detection of NUT protein overexpression; however, because of its rarity and ambiguous protein expression, the correct diagnosis of NUT carcinoma is usually achieved by exclusion, which may lead to pitfalls during diagnosis.

Overestimating specific immunohistochemical expression may complicate the diagnostic process. For example, some NUT carcinomas express neuroendocrine markers, which may result in the misdiagnosis of neuroendocrine carcinoma if stained prior to performing NUT immunohistochemistry.

In the present study, we clarified the challenges of correctly diagnosing NUT carcinoma using immunohistochemistry and comparing the resulting profiles of NUT carcinoma with those of its mimickers, which are encountered in thoracic pathology, particularly basaloid squamous cell carcinoma (BSCC) and small cell lung carcinoma (SCLC).

## Materials and methods

### Case selection

The pathological database of the Cancer Institute Hospital of the Japanese Foundation for Cancer Research (JFCR), Tokyo, was reviewed. Tumors were selected from patients that were diagnosed or treated at the hospital between September 2015 and August 2020. Thoracic and nonthoracic NUT carcinomas, such as tumors originating in the ethmoid sinus, were included because of their high morphological similarity, irrespective of origin. BSCC and SCLC were randomly collected for comparison. The institutional review board approved the study at the JFCR (#2012–1042). The requirement for informed consent was waived and the study was performed following the principles of the Declaration of Helsinki.

### Immunohistochemical staining and method of intensity scoring

Using formalin-fixed, paraffin-embedded tissues, 4-μm-thick slices were cut, stained with antibodies, and analyzed. Insulinoma-associated protein 1 (INSM1) [6, 7], achaete-scute complex-like 1 (ASCL1, MASH1) [8], chromogranin A, synaptophysin, and CD56 were used as conventional neuroendocrine markers, whereas p40 and CK5/6 were used as squamous cell markers. The proto-oncogene bcl-2 (BCL2) was included, which has a significant role in apoptosis inhibition and is highly expressed in SCLC [9, 10].

The following antibodies were used: NUT (rabbit monoclonal, C52B1, dilution 1:50, Cell Signaling, MA, US), chromogranin A (mouse monoclonal, clone DAK-A3, 1:2000; Dako, Carpinteria, CA, US), synaptophysin (mouse monoclonal, clone 27G12, 1:100; Leica Biosystems Newcastle Ltd, UK), CD56 (mouse monoclonal, clone 1B6, 1:50; Leica), INSM1 (mouse monoclonal, clone A-8, diluted 1:500; Santa Cruz, Dallas, TX, US), ASCL1 (mouse monoclonal, clone 24B72D11.1 (anti-MASH1), 1:50; BD Bioscience, Erembodegem, Belgium), p40 (mouse monoclonal, clone BC28, 1:200, Abcam, Cambridge, UK), CK5/6 (mouse monoclonal, clone D5/16B4, 1:200, Millipore, MA, US), TTF-1 (mouse monoclonal, clone 8G7G3/1, 1:100, Dako, Glostrup, Denmark), BCL2 (mouse monoclonal, 124, 1:200, Dako), ProGRP (monoclonal, clone PGCY-9, 1:10000, Fujirebio, Tokyo, Japan), and Ki-67 (mouse monoclonal, clone MIB-1, 1:200, Dako) (Table 1). Immunohistochemistry was carried out using a Bond-III automated immunostainer (Leica Biosystems Melbourne, Melbourne, Australia) and the Bond Polymer Refine Detection Kit as appropriate.

**Table 1:** Details of the antibodies used for immunohistochemistry

Antibody	Company	Clone	Dilution
NUT	Cell Signaling	C52B1	1:50
Chromogranin A	Dako	DAK-A3	1:2000
Synaptophysin	Leica	27G12	1:100
CD56	Leica	1B6	1:50
INSM1	Santa Cruz	A-8	1:500
ASCL1	BD Bioscience	24B72D11.1	1:50
TTF-1	Dako	8G7G3/1	1:100
p40	Abcam	BC28	1:200
CK5/6	Millipore	D5/16B4	1:200
BCL2	Dako	124	1:200
Pro GRP	Fujirebio	PGCY-9	1:10,000
Ki-67	Dako	MIB-1	1:200

The staining results for each antibody were interpreted using an H-score (HS), which was defined by the following equation: Σ (intensity) × (proportion, %). The intensity represents the relative level of tumor cell staining (0, 1, 2, or 3) and the proportion is the percentage of stained tumor cells with an intensity ranging from 0 to 100%. HS varied from 0 to 300. The expression of each antibody in the tumor cells was defined as strongly positive for HS ≥ 100, weakly positive (w +) when HS was ≥ 5, and negative when HS was < 5. For cases in which unstained slides were not available for assessment, 0–10% of stained cells were considered negative and more than 10% were positive.

### Split fluorescence in situ hybridization (FISH) for NUT

Chromosomal translocation of the *NUT* locus was evaluated using FISH. Dual-color split FISH assays were performed on unstained slides (4-μm thick) using *NUT* DNA probes derived from bacterial artificial chromosome (BAC) clones. BAC clones were isolated and used as FISH probes. The names of the BAC clones are available upon request.

### Fusion FISH to detect BRD4-NUT

Formalin-fixed, paraffin-embedded tumor sections underwent deparaffinization, rehydration, and pretreatment. Dual-color FISH probes targeted BRD4 and NUT loci. Post-hybridization, slides were washed and counterstained with DAPI. Fusion *BRD4-NUT* signals were identified as colocalized red and green signals under a fluorescence microscope. Fusion FISH analysis wad performed in case 4 and 8.

### Next generation sequencing to confirm BRD4-NUT fusion

Genomic DNA was extracted from tumor samples and sequenced using Illumina HiSeq X. Libraries were prepared following TruSeq RNA Access Library Prep kit protocols. Sequencing aimed for a minimum 100 × depth. *BRD4-NUT* fusion detection was conducted using FusionCatcher [11].

### Statistical analysis

To analyze statistical significance, a Student’s *t*-test, χ^2^ test, and Fisher’s exact test were used as considered appropriate to evaluate associations among the clinicopathological characteristics. The Kaplan–Meier method was used to estimate survival and the generalized Wilcoxon test was used to determine survival differences. All statistical analyses were done using EZR version 1.40 (Jichi Medical University Saitama Medical Center, Saitama, Japan) and statistical significance was defined as *P* < 0.05.

## Results

### Patient background

Patient details, including age, sex, smoking status, and prognosis, are listed in Table 2. Age at the time of diagnosis was significantly lower in the NUT carcinoma cases (44.5 ± 18.1 years) compared with that in the BSCC and SCLC cases (71.1 ± 9.9 years, *p* = 0.004, 67.8 ± 9.3 years, *p* = 0.007, respectively). Two of the eight NUT carcinoma cases and one case each of BSCC and SCLC were women. Five patients with NUT carcinoma were nonsmokers (5/8, 62.5%); however, all BSCC and SCLC cases were former or current smokers (0/21, nonsmokers 0%, Table 2), which was statistically significant (*p* = 0.0017). The NUT carcinoma specimens were obtained by transbronchial, needle, or excisional biopsies. As with BSCC and SCLC, six and two surgical resection specimens were used for comparison, respectively.

**Table 2:** Clinicopathological background of NUT carcinoma, basaloid squamous cell carcinoma, and small cell carcinoma

	Age	Sex	Site	Smoking	Smoking index	Specimen	Time (days)	Prognosis	Initial diagnosis
NUT carcinoma (*n* = 8)									
1	67	M	Lung	Former	450	Biopsy	32	Dead	Small cell carcinoma
2	64	M	Pleura	Former	430	Biopsy	71	Dead	Malignant cells (pleural effusion)
3	46	M	Lung	Former	220	Biopsy	73	Dead	Small cell carcinoma or squamous cell carcinoma, p/d
4	46	M	Maxillary sinus	Never	0	Biopsy	139	Dead	Nonepithelial tumor
5	47	M	Ethmoid sinus	Never	0	Biopsy	2167	Alive	Squamous cell carcinoma
6	49	F	Ethmoid sinus	Never	0	Biopsy	904	Alive	Squamous cell carcinoma
7	15	F	Lung	Never	0	Biopsy	272	Alive	NUT carcinoma
8	22	M	Maxillary sinus	Never	0	Biopsy	530	Alive	Squamous cell carcinoma
average age ± SD : 44.5 ± 18.1									
Basaloid squamous cell carcinoma (*n* = 8)									
1	81	M	Lung	Former	435	Resection	570	Alive	
2	54	M	Lung	Former	560	Biopsy	1986	Dead	
3	65	M	Lung	Current	780	Resection	2649	Alive	
4	64	M	Lung	Current	820	Resection	2133	Alive	
5	82	M	Lung	Current	1000	Resection	2519	Alive	
6	78	M	Lung	Former	800	Biopsy	713	Dead	
7	68	M	Lung	Former	1760	Resection	1146	Dead	
8	77	F	Lung	Former	1000	Resection	1142	Alive	
average age ± SD : 71.1 ± 8.4									
Small cell carcinoma (*n* = 13)									
1	63	M	Lung	Former	1500	Biopsy	235	Dead	
2	69	M	Lung	Former	900	Resection	576	Dead	
3	62	M	Lymph node	Current	1600	Biopsy	1143	Dead	
4	71	M	Lymph node	Current	2040	Biopsy	119	Dead	
5	83	M	Lung	Former	1120	Biopsy	63	Alive	
6	49	M	Lymph node	Former	1160	Biopsy	708	Dead	
7	81	F	Lung	Current	470	Biopsy	642	Dead	
8	61	M	Lymph node	Former	820	Biopsy	713	Dead	
9	66	M	Lung	Former	840	Resection	343	Dead	
10	64	M	Lung	Former	860	Biopsy	653	Alive	
11	76	M	Liver meta	Former	1020	Biopsy	391	Dead	
12	75	M	Lymph node	Former	1000	Biopsy	1085	Alive	
13	62	M	Lymph node	Current	840	Biopsy	251	Dead	
average age ± SD : 67.8 ± 9.0									

### Histological comparison of NUT carcinoma, BSCC, and SCLC

**Fig. 1:**
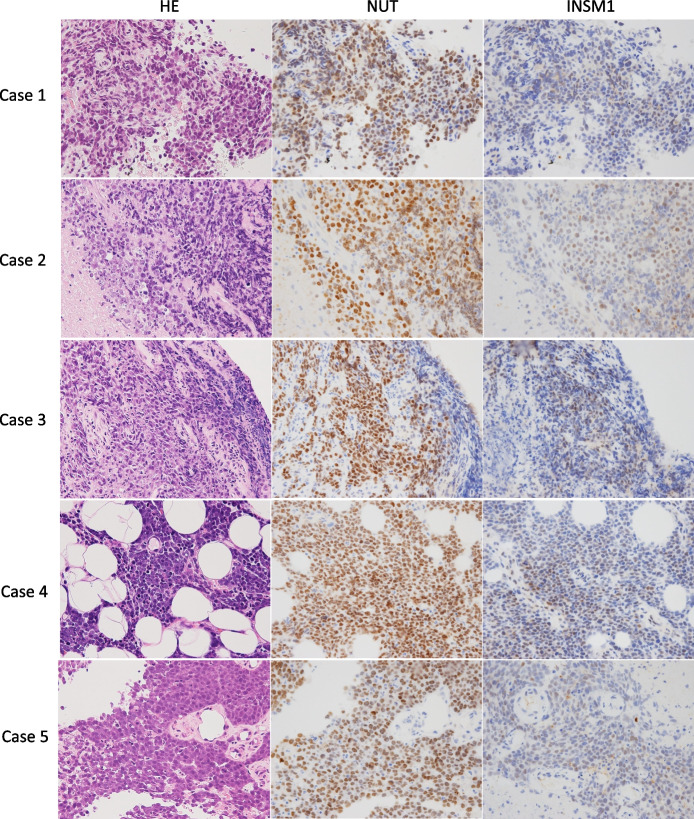
Histological findings from hematoxylin & eosin staining and immunohistochemistry (NUT and INSM1) of NUT carcinoma. Positive nuclear staining of INSM1 was readily detected in tumor cells, although the staining intensity was generally weak

**Fig. 2:**
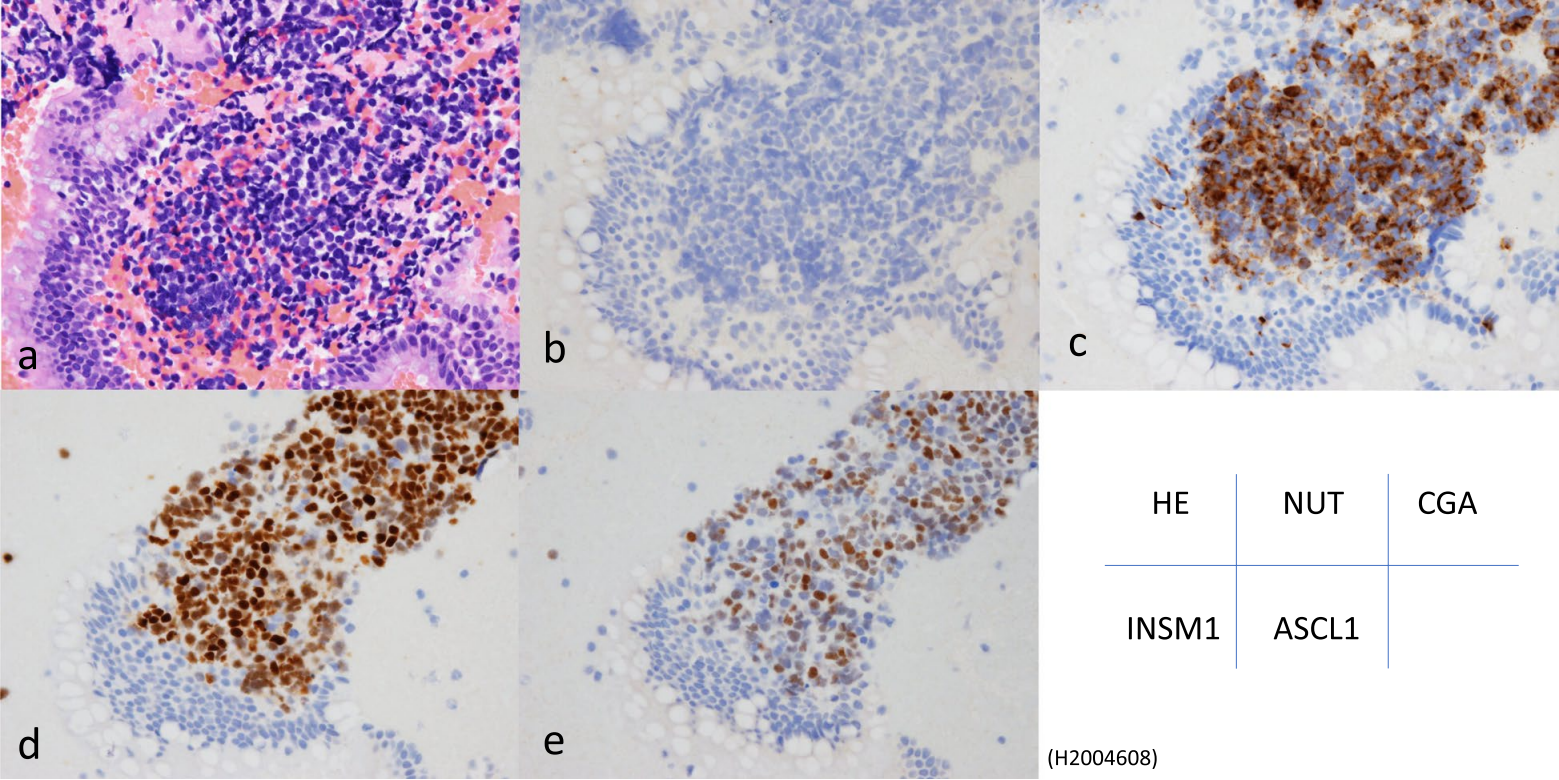
Representative histology and immunohistochemistry of small cell lung carcinoma. Hematoxylin & eosin (**a**), NUT (**b**), chromogranin A showing cytoplasmic positivity in tumor cells (**c**). Both INSM1 (**d**) and ASCL1 (**e**) were positive in the nuclei

**Fig. 3:**
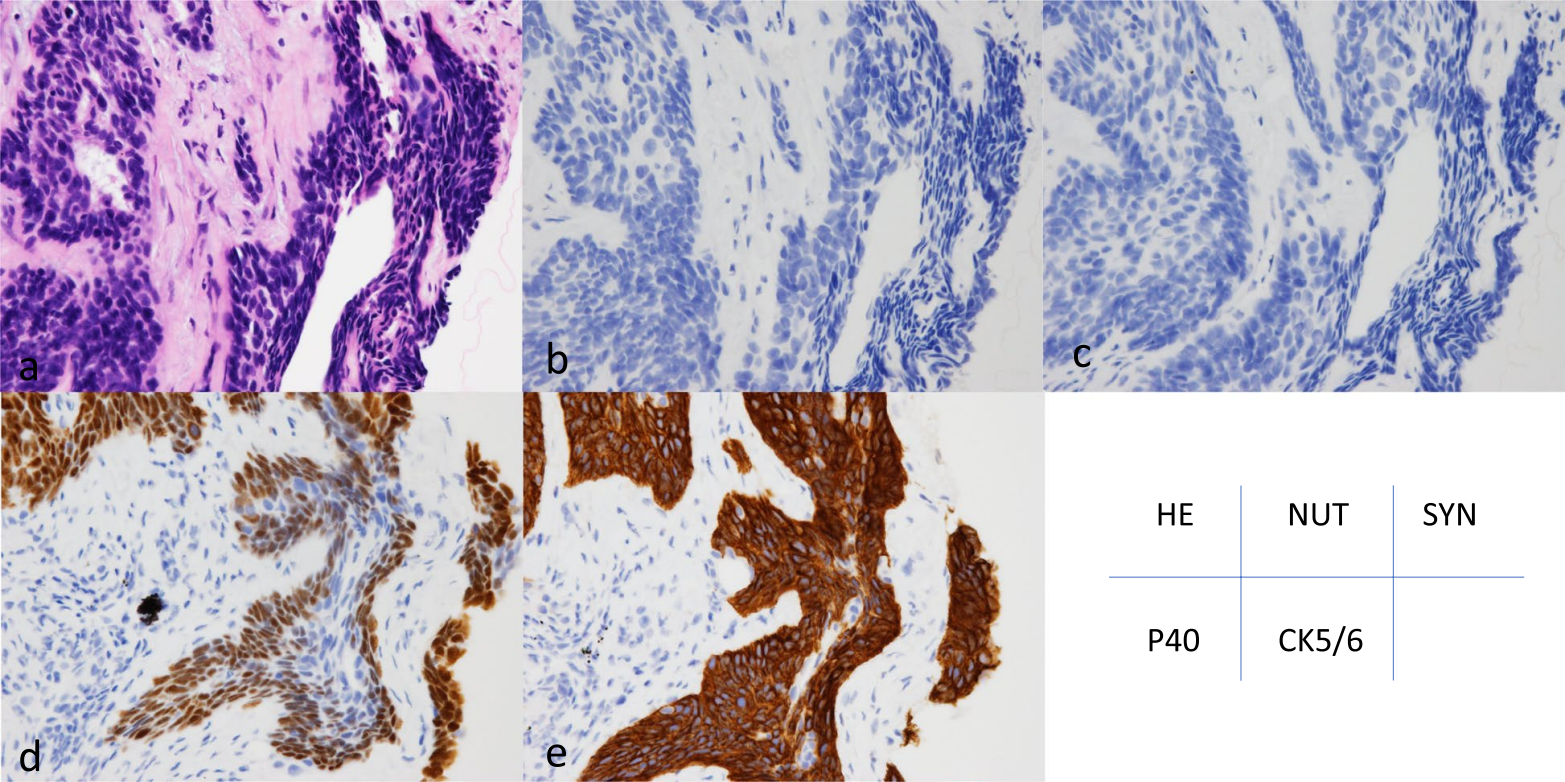
Representative histology and immunohistochemistry of basaloid squamous cell carcinoma. Hematoxylin & eosin (**a**), NUT (**b**), synaptophysin (**c**), P40 was positive for tumor cells in the basal layer (**d**) and CK5/6 (**e**) strong and diffuse staining in the cytoplasm

NUT carcinoma, BSCC, and SCLC exhibited a slightly similar morphology, in which they were composed of monomorphic tumor cells proliferating in a solid or sheet-like structure (Figs. 1, 2 and 3). Initially, only one case was correctly diagnosed as NUT carcinoma, whereas three were diagnosed as squamous cell carcinoma and two were considered SCLC, one with malignant cells and one a non-epithelial tumor. Of the eight NUT carcinoma cases, abrupt keratinization was observed in only three (3/8 and Cases 5, 6, and 8, Table 3).

**Table 3:** Immunohistochemical profiles of NUT carcinoma, basaloid squamous cell carcinoma, and small cell carcinoma

Case	NUT	CGA	SYN	CD56	TTF-1	P40	CK5/6	INSM1	ASCL1	BCL2	ProGRP	Ki-67	Abrupt keratinization	NUT split FISH	Fusion variant
NUT carcinoma (*n* = 8)															
1	+	-	+ ^a^	-^a^	-	+ ^a^	-	w +	-	-	-	NE	-	NE	NE
2	+	-	-	-	-	-	-	w +	-	w +	-	90	-	positive	NE
3	+	-	-	-	-	w +	w +	w +	-	-	-	80	-	positive	NE
4	+	-	w +	-	-	-	-	w +	-	+	-	50	-	positive	*BRD4-NUT*
5	+	-	-	-	-	+	+	w +	-	-	-	80	+	positive	NE
6	+ ^a^	-	-	-	NE	NE	+ ^a^	NE	NE	NE	NE	NE	+	positive	NE
7	+ ^a^	NE	NE	NE	NE	+ ^a^	NE	NE	NE	NE	NE	NE	-	NE	NE
8	+	-	-	-	-	+	+	w +	-	w +	-	70	+	positive	*BRD4-NUT*
Basaloid squamous cell carcinoma (*n* = 8)															
1	-	-	-	-	-	+	+	-	-	+	NE	10			
2	-	-	-	-	-	+	+	-	-	+	NE	20			
3	-	-	-	-	-	+	+	-	-	+	NE	30			
4	-	-	-	-	-	+	+	-	-	+	NE	10			
5	-	-	-	-	-	+	+	-	-	+	NE	90			
6	-	-	-	w +	-	w +	+	-	-	+	NE	60			
7	-	-	-	+	-	+	+	-	-	+	NE	70			
8	-	-	-	-	-	+	+	w +	-	+	NE	80			
Small cell carcinoma (*n* = 13)															
1	-	+	+	+	+	-	-	+	+	+	-	90			
2	-	w +	+	+	+	-	-	+	+	+	+	90			
3	-	w +	+	+	+	w +	-	+	w +	w +	-	85			
4	-	-	+	+	+	-	-	+	w +	+	+	90			
5	-	-	+	+	+	-	-	+	-	+	-	95			
6	-	w +	+	+	+	-	-	+	+	+	w +	70			
7	-	w +	+	+	+	-	-	+	+	+	+	95			
8	-	+	+	+	+	-	-	+	+	+	w +	95			
9	-	w +	+	+	+	-	-	+	w +	+	w +	90			
10	-	-	w +	+	+	-	-	+	+	+	-	95			
11	-	-	-	-	-	-	-	w +	-	+	-	95			
12	-	w +	+	+	+	-	-	+	+	+	w +	80			
13	-	+	+	+	+	-	-	+	+	+	-	100			

*CGA* Chromogranin A, SYN Synaptophysin, *NE* Not examined

^a^stained slides were not available. positive cells; 1%–10% (-), more than 10% ( +) positive ( +): H-score was ≥ 100, weakly positive (w +) when the H-score was between 5 and 100 and negative when the H-score was < 5

#### Results of immunohistochemistry with NUT

All NUT carcinomas were positive for NUT as determined by immunohistochemistry (Tables 3, 4 and Fig. 1), whereas none of the BSCC and SCLC were positive.

**Table 4:** Expression levels of each antibody evaluated using H-scores

Case	NUT	CGA	SYN	CD56	TTF-1	P40	CK5/6	INSM1	ASCL1	BCL2	ProGRP
NUT carcinoma (*n* = 8)											
1	270	0	NE	NE	0	NE	0	40	0	0	0
2	285	0	3	0	0	0	0	50	0	20	0
3	300	0	0	0	0	40	60	60	0	2	0
4	285	0	80	0	0	0	0	75	0	180	0
5	285	0	0	0	0	300	285	50	0	0	0
6	NE	0	0	0	NE	NE	NE	NE	NE	NE	NE
7	NE	NE	NE	NE	NE	NE	NE	NE	NE	NE	NE
8	300	0	0	3	0	300	220	50	0	30	0
Basaloid squamous cell carcinoma (*n* = 8)											
1	0	0	0	0	0	300	300	0	0	300	0
2	0	0	0	0	0	210	300	0	0	100	0
3	0	0	0	0	0	300	300	5	0	300	0
4	0	0	0	0	0	300	300	0	0	300	0
5	0	0	0	0	0	300	300	0	0	300	0
6	0	0	0	20	0	30	160	0	0	300	0
7	0	0	0	150	0	300	270	0	0	300	0
8	0	1	0	0	0	300	300	60	0	300	0
Small cell lung carcinoma (*n* = 13)											
1	0	10	180	300	300	0	0	160	160	300	0
2	0	5	200	300	300	0	0	255	300	300	140
3	0	30	100	300	180	80	0	300	5	5	0
4	0	0	285	300	300	0	0	120	90	270	100
5	0	0	180	300	300	0	0	300	0	285	0
6	0	5	80	300	190	0	0	270	270	300	20
7	0	10	285	300	300	0	0	285	140	300	120
8	0	120	150	300	240	0	0	255	285	300	50
9	0	55	300	300	300	0	0	120	90	300	25
10	0	0	60	300	300	0	0	150	240	300	0
11	0	0	0	0	0	0	2	30	0	300	0
12	0	20	300	300	300	0	0	285	160	300	50
13	0	270	300	160	300	0	0	120	270	300	0

For other abbreviations and H-score, see text

*CGA* Chromogranin A, *SYN* Synaptophysin, *NE* Not examined

#### Results of the three conventional neuroendocrine markers as well as INSM1 and ASCL1

Synaptophysin was positive by immunohistochemical analysis for some NUT carcinoma cases; however, no cases were positive for chromogranin A or CD56. INSM1 is a promising marker for SCLC [6] and it was positive in all six NUT carcinoma cases available for staining as determined by immunohistochemistry (Fig. 1). Although the positive staining ratio was high, the intensity was weak (1 +) in contrast to the strong expression observed in SCLC (Table 3). ASCL1, which is also considered a useful marker for pulmonary small cell carcinoma, was negative in the NUT carcinomas.

#### Results of immunohistochemistry for TTF-1, p40, and CK5/6

All of the NUT carcinomas were negative for TTF-1 (0%,) whereas five were weakly or diffusely positive for p40. Two cases with abrupt keratinization exhibited strong positivity for one or both squamous cell markers; however, all BSCC cases, excluding one, were strongly positive for both p40 and CK5/6 (Tables 3 and 4).

#### Results of immunohistochemistry with BCL2 and ProGRP

The BCL2 oncoprotein has a unique role in the inhibition of programmed cell death (apoptosis), which results in tumorigenesis and chemoresistance. Immunohistochemical analysis of BCL2 revealed that it was upregulated in most small cell carcinomas [9]. All cases of BSCC and SCLC except one exhibited strong positive staining for BCL2. Interestingly, only two cases of NUT carcinoma were positive. ProGRP is a serum biomarker of small cell carcinoma and its protein expression was reported to be higher in SCLC tissues compared with control tissues [12]. ProGRP positivity was only observed in SCLC cases (7/13 = 54%) (Tables 3 and 4).

#### Results of Split FISH Analysis on NUT and BRD4-NUT Fusion Detection

**Fig. 4:**
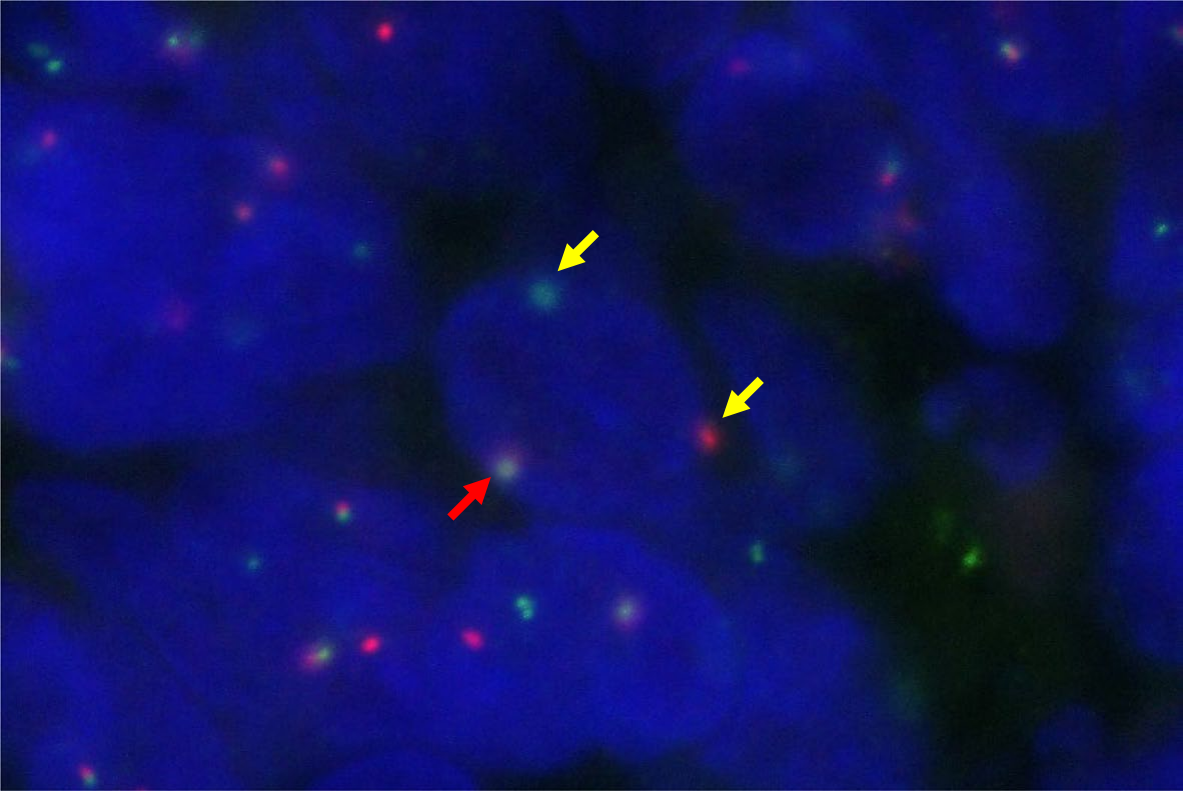
The presence of two pairs of fused green and red signals was considered a normal finding (red arrow). In contrast, one fused red/green signal and one separate red and green signal are evident in the translocation-positive nuclei (yellow arrow)

Six NUT carcinoma that have FISH testing results all showed NUT translocation based on a split FISH analysis, as represented by isolated green and red signals flanking the *NUT* gene (Fig. 4). In Cases 4 and 8 *BRD4-NUT* fusion was confirmed by characterized by the co-localization of BRD4 and NUT-specific fluorescent signals.

### Next generation sequencing to detect BRD4-NUT fusion

HiSeq X sequencing technology coupled with FusionCatcher software identified the presence of the BRD4-NUTM1 fusion gene in case 8. The resulting sequence analysis revealed the following fusion junction:GGAGAGCTCCAGTGAGTCCAGCTCCTCTGACAGCGAAGACTCCGAAACA*GTGACCGCTCCAAAATTTCCAAGGACGTTTATGAGAACTTCCGTCAGTGG. Notably, the fusion was found to be out-of-frame, indicating a potential disruption in the normal reading frame of the genes involved.

### Comparison of cancer-specific survival for NUT carcinoma, SCLC, and BSCC

**Fig. 5:**
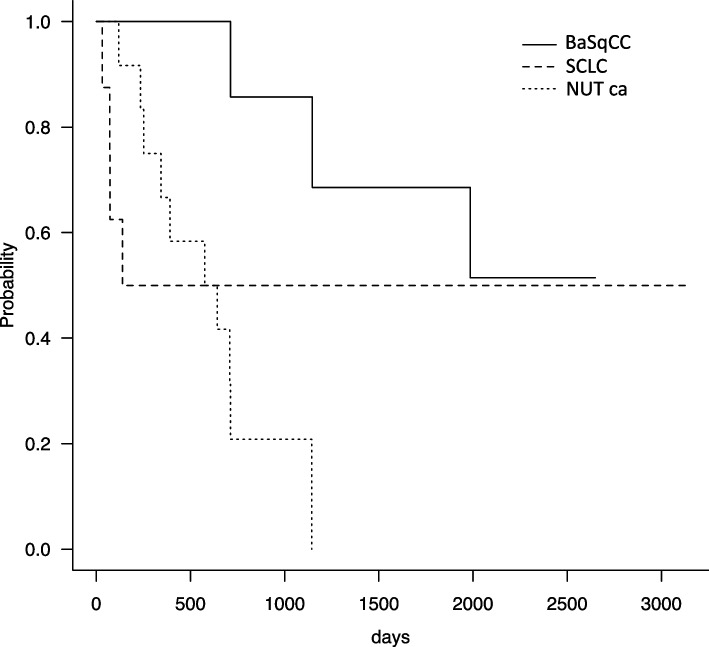
Cancer-specific survival curves of NUT carcinoma, basaloid squamous cell carcinoma, and small cell carcinoma. The 1-year prognosis of NUT carcinoma was remarkably poor, although there may exist a subgroup of NUT carcinomas which have a better prognosis. The difference in long-term prognosis between BSCC or SCLC was statistically significant (*p* = 0.0032)

Figure 5 shows the overall survival curves for the three groups. Four NUT carcinoma patients died within 5 months after diagnosis and early prognosis was considerably poor compared with that of the other two tumor types. However, the other four NUT carcinoma patients exhibited exceptional long-term survival; thus, there may be a subgroup of NUT carcinomas which have a good prognosis. Therefore, the statistical differences between NUT carcinoma and the other two diseases were insignificant (generalized Wilcoxon test: NUT vs. SCLC, *p* = 0.79; NUT vs. BSCC, *p* = 0.19; BSCC vs. SCLC, *p* = 0.0032).

## Discussion

NUT carcinomas primarily exhibit an undifferentiated appearance both morphologically and immunohistochemically. The differential diagnosis of NUT carcinoma includes a wide variety of diseases, which may occasionally lead to an incorrect diagnosis. Immunohistochemistry is a reliable diagnostic tool for this tumor type, even when applied to small biopsy specimens; however, ambiguous immunohistochemical expression in NUT carcinomas can result in an incorrect diagnosis. Occasionally, NUT carcinomas present as a characteristic squamous-differentiated morphology, namely abrupt keratinization and some are defined as a subtype of squamous cell carcinoma [13], although squamous histology is not always apparent. Moreover, there is limited data with respect to neuroendocrine and squamous differentiation marker expression in NUT carcinomas and the results of previous studies have been inconclusive. Therefore, in the present study, we carried out a comprehensive immunohistochemical analysis of NUT carcinoma under similar conditions to identify the diagnostic pitfalls.

Several studies have been done on the immunohistochemical expression of NUT carcinomas (Table 5) [13–17]; however, the antibodies used varied depending on the site of origin and the possible differential diagnoses were diverse. In the present study, we focused on antibodies that can discriminate the major mimickers of thoracic origin, which include SCLC and BSCC. Therefore, the analysis of neuroendocrine and squamous cell markers is important. Sholl et al. [15] reported two cases of NUT carcinomas out of nine that were positive for synaptophysin and CD56, respectively, in the largest series of cases examined at a single institution, although the percentage (2/9 = 22%) was not high. Moreover, a recent study described a case of NUT carcinoma exhibiting positive synaptophysin expression that was misdiagnosed as a large cell neuroendocrine carcinoma [18]. These results suggest that placing a high priority on a specific antibody can lead to an incorrect diagnosis. Abrupt keratinization is a well-known feature of NUT carcinoma, which may represent an aggressive subtype of squamous cell carcinoma [19]. Unexpectedly, the positive staining rates for both p40 and CK5/6 in NUT carcinomas were variable and relatively low in our panel. One patient with abrupt keratinization exhibited strong positivity for both markers. Although p63 has been reported to have a high rate of positivity (Table 4), it has low specificity [20].

**Table 5:** Summary of the immunohistochemical studies of NUT carcinoma in the literature

Author	Site	N	CGA	SYN	CD56	TTF-1	p40	p63	CK5/6	pan keratin
Evans (2012)	Mediastinum	4	0/2 (0%)	0/3 (0%)	0/1 (0%)	0/3 (0%)	-	0/1 (0%)	-	-
Sholl (2015)	Lung	9	0/5 (0%)	1/6 (17%)	1/5 (20%)	3/8 (38%)	-	6/9 (67%)	-	-
Mao^a^ (2019)	Lung	11	0/4 (0%)	2/7 (29%)	1/3 (33%)	1/6 (17%)	-	7/7 (100%)	0/2 (0%)	5/8 (63%)
Agaimy^a^ (2018)	Salivary Gland	10	0/8 (0%)	2/9 (22%)	2/9 (22%)	-	-	8/8 (100%)	-	8/8 (100%)
Lee (2019)	Head and neck	4	-	-	0/4 (0%)	-	3/3 (100%)	4/4 (100%)	-	4/4 (100%)
**Our study** **(2023)**	**Various**	**8**	**0/7** **(0%)**	**2/7** **(29%)**	**0/7** **(0%)**	**0/6** **(0%)**	**5/7** **(71%)**	**-**	**4/7** **(57%)**	-

^a^review article, -; no data, *CGA* Chromogranin A, *SYN* Synaptophysin

Recent studies have indicated that INSM1 is a sensitive and specific marker for SCLC [6, 7, 21–24]. It may be useful when other traditional neuroendocrine markers are negative. Only a few studies have considered INSM1 expression in NUT carcinoma [22, 25]. In the present study, all NUT carcinoma cases were positive for INSM1, which is in contrast to the results (0/5) reported by Tsai et al. [22], even though the same antibody was used. We consider the possibility of variations in sample size, technical aspects and biological variability among tumors, which could inherently cause differences in antigen expression. In the present study, it must be emphasized that the ratio of positive cells was relatively high (HS: 40–75%), although the staining intensity was weak (Table 4 and Fig. 1). Thus, positive INSM1 expression with an ambiguous morphology in small specimens may be a deceptive phenotype that requires careful consideration. Moreover, the expression of ASCL1, a transcription factor involved in the development of pulmonary neuroendocrine cells [6, 26], was not detected in NUT carcinomas. This was in contrast to the observed simultaneous expression of INSM1 and ASCL1, which was prevalent in the SCLC cases (Table 3). Discrepancies between the two neuroendocrine markers may also be useful for avoiding diagnostic traps. Although BCL2 and ProGRP are potential markers for SCLC [9, 10, 27], their specificity and sensitivity were lower in NUT carcinomas compared with that in SCLC.

NUT carcinoma should be suspected in patients without a history of smoking or minimal smoking with advanced disease during their initial diagnosis [18]. Most patients with NUT carcinoma generally have a short life expectancy; however, differences between prognostic risk groups defined by clinical and molecular profiles are statistically significant. In other words, primaries outside of the thorax with *non-BRD4-NUT* fusions (such as *BRD3-NUT* or *NSD3-NUT*) are associated with the best prognosis. A possible explanation for the worse prognosis in thoracic primaries is that they are less accessible and often present at a more advanced stage, or they may have a different cell biology [28]. In the present study, overall survival curves showed that non-thoracic origin had a better prognosis, though the difference was not statistically significant (data not shown).

Prompt and accurate diagnoses of lung tumors are essential to develop optimal treatments. Because most mimickers are smoking-related carcinomas, such as SCLC and BSCC, because more than half of the NUT carcinoma cases in this study were nonsmokers, may be a clue to consider the possibility of NUT carcinoma. In Case 1, small cell carcinoma was initially diagnosed. However, the neoplasm was refractory to standard therapeutic interventions, and further evaluation demonstrated immunohistochemical positivity for the NUT immunohistochemistry. For Case 2, histopathological assessment failed to identify a definitive carcinoma subtype and its origin, yet a thorough immunohistochemical analysis indicated positive staining for NUT. In Case 3, although initially characterized as small cell carcinoma, the presence of marked neutrophilic infiltration, indicative of NUT carcinoma, necessitated additional immunohistochemical investigation. In Case 4, a never smoker, an extensive immunohistochemical workup was previously conducted, assessing epithelial, mesenchymal, neurogenic, and melanocytic markers; only cytokeratin returned positive results. The high nuclear-to-cytoplasmic (N/C) ratio suggested small cell carcinoma, yet the presence of prominent nucleoli rendered the morphology indeterminate. Cases 5, 6, and 8, all never-smokers, were originally classified as squamous cell carcinoma. However, the abrupt keratinization observed prompted the application of immunohistochemical staining, fluorescence in situ hybridization (FISH), and next-generation sequencing to verify NUT carcinoma. We propose a more systematic use of NUT-specific immunohistochemistry in cases where the diagnosis is uncertain, especially when dealing with poorly differentiated tumors that present with ambiguous morphology. We also emphasize the need for a high index of suspicion in atypical presentations, particularly in younger patients and those without a significant smoking history, as highlighted by our study's findings.

In conclusion, NUT carcinomas exhibit a characteristic immunophenotype when squamous cell and neuroendocrine differentiation markers were examined. Our results indicate that reliable squamous cell markers are expressed variably among NUT carcinoma cases. The positive ratio for INSM1 in NUT carcinoma was relatively high; however, the intensity was low without exception. These results are the basis for the difference with BSCC and SCLC, which are sometimes indistinguishable based on morphology alone. Our findings underscore the necessity of conducting an exhaustive immunohistochemical evaluation to distinguish NUT carcinoma in cases presenting with ambiguous morphology and atypical clinical features.

## Data Availability

The datasets generated and/or analyzed in the present study are not publicly available, but are available from the corresponding author upon reasonable request.
